# Gut Microbiota: An Important Player in Type 2 Diabetes Mellitus

**DOI:** 10.3389/fcimb.2022.834485

**Published:** 2022-02-15

**Authors:** Zheng Zhou, Bao Sun, Dongsheng Yu, Chunsheng Zhu

**Affiliations:** ^1^Department of Chinese Medicine, The First Affiliated Hospital of Zhengzhou University, Zhengzhou, China; ^2^Department of Pharmacy, The Second Xiangya Hospital, Central South University, Changsha, China; ^3^Institution of Clinical Pharmacy, Central South University, Changsha, China

**Keywords:** gut microbiota, glucose metabolism, insulin resistance, type 2 diabetes mellitus, pathogenesis

## Abstract

Type 2 diabetes mellitus (T2DM) is one of the common metabolic diseases in the world. Due to the rise in morbidity and mortality, it has become a global health problem. To date, T2DM still cannot be cured, and its intervention measures mainly focus on glucose control as well as the prevention and treatment of related complications. Interestingly, the gut microbiota plays an important role in the development of metabolic diseases, especially T2DM. In this review, we introduce the characteristics of the gut microbiota in T2DM population, T2DM animal models, and diabetic complications. In addition, we describe the molecular mechanisms linking host and the gut microbiota in T2DM, including the host molecules that induce gut microbiota dysbiosis, immune and inflammatory responses, and gut microbial metabolites involved in pathogenesis. These findings suggest that we can treat T2DM and its complications by remodeling the gut microbiota through interventions such as drugs, probiotics, prebiotics, fecal microbiota transplantation (FMT) and diets.

## Introduction

Type 2 diabetes mellitus (T2DM) is a progressive metabolic disease characterized by pancreatic β-cell dysfunction and peripheral insulin resistance, leading to defects in glucose metabolism and chronic low-grade inflammation. The development of this chronic disease is closely related to both genetic and environmental factors, and certain environmental factors, such as caloric intake, nutrient composition, ambient air pollution and physical inactivity, are important reasons for the continuous increase in its prevalence (Kahn et al., 2014; Kang et al., 2022). It was reported that more than 460 million adults worldwide had diabetes in 2019, and this number would rise to 700 million by 2045 (Saeedi et al., 2019). Since T2DM contributes to the occurrence of harmful macrovascular and microvascular outcomes (Sehgal et al., 2021; Zeng et al., 2021; Zhang X. et al., 2021; Zhao et al., 2021), it not only brings physical and mental pain to patients, but also leads to a major medical economic burden. Therefore, it is of great significance to explore the novel pathogenesis and treatment of T2DM.

The gut microbiota is considered to be a complex ecosystem in the gastrointestinal tract environment, which is composed of bacteria, archaea, fungi, viruses and protozoa (Wang et al., 2021). Growing evidence indicates that the gut microbiota plays a vital role in human health, and its dysbiosis is associated with a variety of pathological processes (Crittenden and Goepp, 2021; Han et al., 2021; Mayneris-Perxachs et al., 2021). Notably, many studies have linked the gut microbiota to T2DM (Kashtanova et al., 2018; Takagi and Naito, 2020). For example, researchers found that 43 bacterial taxa were significantly different between Chinese obese individuals with T2DM and healthy people by LEfSe analysis, and *Acidaminococcales*, *Bacteroides plebeius* and *Phascolarctobacterium* sp.*CAG207* might be potential biomarkers for T2DM (Wang T.Y. et al., 2020). Here, we summarize the characteristics of the gut microbiota in T2DM population, T2DM animal models, and diabetic complications, as well as the molecular mechanisms linking host and the gut microbiota in T2DM, which may provide new ideas for the treatment of T2DM.

## Gut Microbiota and T2DM

**Figure 1:**
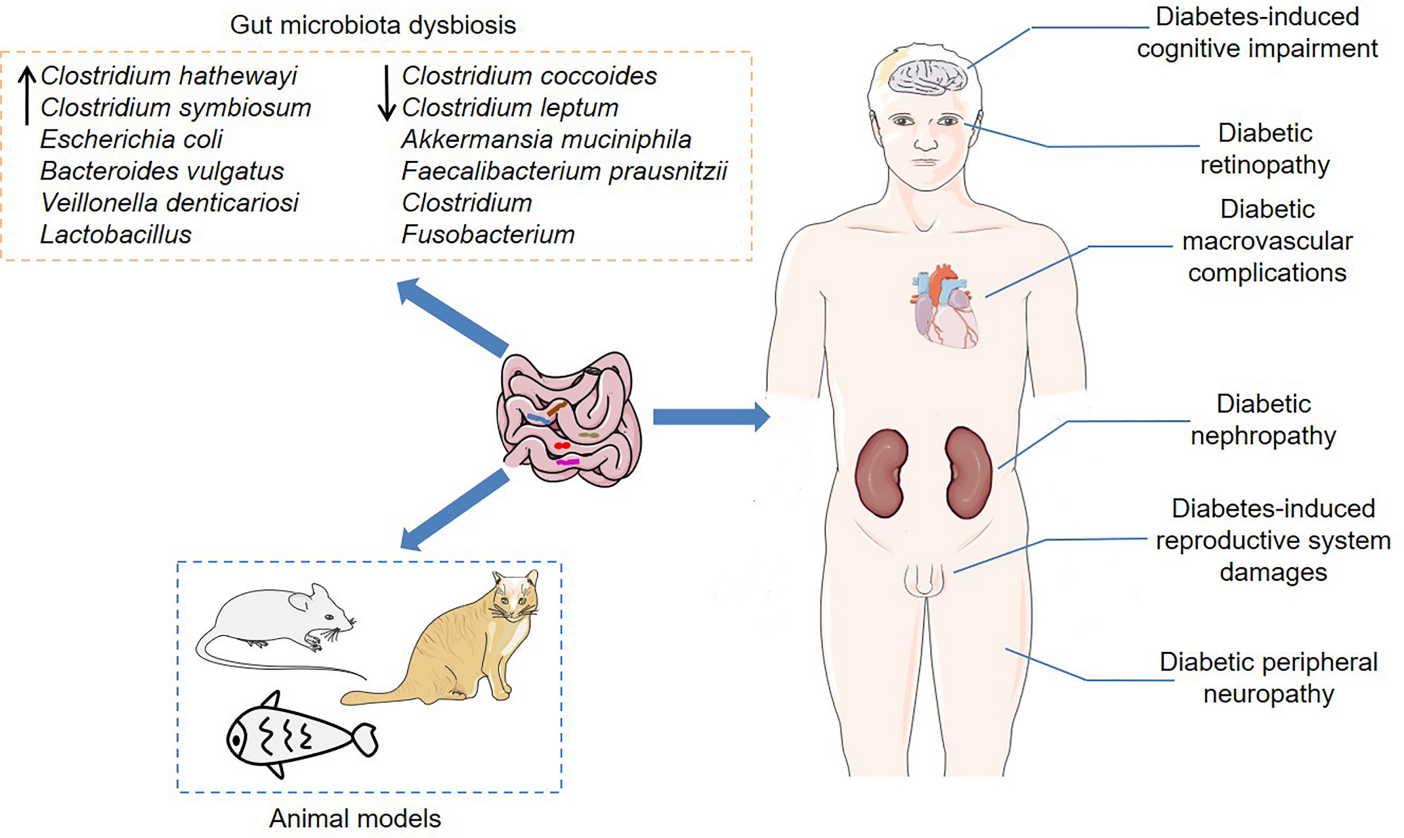
The association between the gut microbiota and T2DM. Dysbiosis of the gut microbiota has been demonstrated not only in T2DM populations, but also in certain animal models, including mice, cats and zebrafish. Furthermore, the gut microbiota is closely associated with various diabetic complications, such as diabetic nephropathy, diabetes-induced cognitive impairment, diabetic retinopathy, and diabetic peripheral neuropathy.

The gut microbiota is involved in obesity, non-alcoholic fatty liver (NAFL), insulin resistance and chronic inflammation, which are related to the development of T2DM (Saad et al., 2016; Mouries et al., 2019; Lee J.Y. et al., 2021). Obese individuals generally have a low abundance of intestinal bacteria (Le Chatelier et al., 2013). Comprehensive analysis of targeted metagenomics and metabolomics found that Italian NAFL patients showed lower levels of *Oscillospira* and higher levels of 2-butanone and 1-pentanol compared to healthy controls, indicating that the gut microbiota might play an important role in liver steatosis (Del Chierico et al., 2017). Moreover, Horne et al. discovered that a high-fat and high-fructose diet could change the composition of the gut microbiota in Syrian hamsters, leading to dyslipidemia and hepatomegaly (Horne et al., 2020). Among them, *Ruminiclostridium 9* and *Tyzzerella* were positively correlated with fasting cholesterol levels, while *Tyzzerella* and *Ruminococceace NK4A214 group* were positively correlated with fasting triglyceride levels. These findings suggest that the gut microbiota is closely linked to a cluster of metabolic disorders. Of note, the gut microbiota has been demonstrated to be altered in diabetic populations and animal models, implying that it may be an important participant in the pathogenesis of T2DM (**Figure 1**).

### Gut Microbiota Dysbiosis in T2DM Population

To date, substantial evidence of gut microbiota dysbiosis has been found in T2DM individuals (**Table 1**). Previous research found that the gut microbiota was moderately dysregulated in Chinese T2DM patients (Qin et al., 2012). Specifically, T2DM patients showed an increase in multiple pathogenic bacteria, such as *Clostridium hathewayi*, *Clostridium symbiosum* and *Escherichia coli*, while healthy controls had a high abundance of butyrate-producing bacteria. Another study confirmed that European women with T2DM had a higher abundance of four *Lactobacillus* species and a lower abundance of five *Clostridium* species compared with the normal glucose tolerance individuals (Karlsson et al., 2013). Importantly, *Lactobacillus* species were positively correlated with fasting glucose and glycosylated hemoglobin (HbA1c), while *Clostridium* species were negatively correlated with fasting glucose, HbA1c and plasma triglycerides, suggesting that these bacterial taxa might be related to the development of T2DM. Similarly, the levels of *Lactobacillus* were significantly increased, while the levels of *Clostridium coccoides* and *Clostridium leptum* were significantly decreased in newly diagnosed T2DM patients (Chen et al., 2019). Notably, Shih et al. studied the characteristics of the intestinal microbiota of patients with refractory T2DM (RT2D), whose HbA1c still increased by at least 8% under treatment (Shih et al., 2020). Compared with T2DM controls, RT2D patients showed higher abundance of *Bacteroides vulgatus* and *Veillonella denticariosi*, and lower abundance of *Akkermansia muciniphila* and *Fusobacterium*. Of these, the relative levels of *A. muciniphila* were negatively correlated with HbA1c.

**Table 1:** Major findings from the studies of patients with T2DM.

Patient cohort	Strategy	Characteristics	Main observations	References
T2DM	Whole-genome metagenomics shotgun	Chinese, mean age 47.0±4.5 years	*Acidaminococcales*, *Bacteroides plebeius* and *Phascolarctobacterium* sp. CAG207 might be potential biomarkers for T2DM	Wang T.Y. et al., 2020
T2DM	A two-stage metagenome-wide association study based on deep shotgun sequencing	Chinese, aged 13–86 years	T2DM patients showed an increase in multiple pathogenic bacteria, while healthy controls had a high abundance of butyrate-producing bacteria	Qin et al., 2012
T2DM	Whole-genome metagenomics shotgun	70-year-old European women	European women with T2DM had a higher abundance of four *Lactobacillus* species and a lower abundance of five *Clostridium* species	Karlsson et al., 2013
Newly diagnosed T2DM	16S rRNA sequencing	Taiwanese individuals, mean age 51±12 years	The levels of *Lactobacillus* were increased, while the levels of *Clostridium coccoides* and *Clostridium leptum* were decreased in newly diagnosed T2DM patients	Chen et al., 2019
RT2D	16S rRNA sequencing	Taiwanese individuals, mean age 64.37±2.194 years	RT2D patients showed higher abundance of *Bacteroides vulgatus* and *Veillonella denticariosi*, and lower abundance of *Akkermansia muciniphila* and *Fusobacterium*	Shih et al., 2020
Prediabetes	16S rRNA sequencing	Danish, aged 57–68 years	The abundance of *Clostridium* genus and *A. muciniphila* was decreased in Danish patients with prediabetes	Allin et al., 2018
Prediabetes	Whole-genome metagenomics shotgun	Chinese, mean age 61.75 ± 9.09 years	The abundance of MLGs from the *Clostridia* class and *Faecalibacterium prausnitzii* was lower, while that from *E. coli*, *Streptococcus salivarius*, and *Eggerthella* sp. was higher in prediabetics	Zhong et al., 2019
DN	16S rRNA sequencing	Chinese, mean age 52.93 ± 9.98 years	*Escherichia-Shigella* and *Prevotella_9* genera could distinguish DN patients from T2DM without renal disease patients	Tao et al., 2019
DN	16S rRNA sequencing	Chinese, mean age 60.86 ± 5.69 years	Patients with stage IV DN had a higher abundance of *Haemophilus* and *Lachnospiraceae_UCG-004* compared with patients with stage III DN	Du et al., 2021
DCI	16S rRNA sequencing	Chinese, mean age 69.41 ± 4.16 years	DCI patients showed a decreased abundance of *Bifidobacterium*, *Tenericutes* and *unrank-RF39*, as well as an increased abundance of *Peptococcus* and *unrank-Leuconostocaceae*	Zhang et al., 2021a
DR	16S rRNA sequencing	Chinese, mean age 60.28 ± 10.5 years	*Pasteurellaceae* might be used as a non-invasive biomarker for the diagnosis of DR	Huang et al., 2021

Prediabetes is a pathological condition in which blood glucose levels are higher than normal but lower than diabetes thresholds, and individuals with prediabetes are at increased risk of T2DM (Tabák et al., 2012). Intriguingly, prediabetics also have aberrant gut microbiota (Zhou et al., 2019a; Chávez-Carbajal and Pizano-Zárate, 2020). Compared with individuals with normal glucose regulation, the most significant feature of the gut microbiota in Danish patients with prediabetes was the decreased abundance of *Clostridium* genus and *A. muciniphila* (Allin et al., 2018). Zhong et al. observed that there was no significant difference in microbial gene-based richness between treatment-naïve T2DM, prediabetes and normal glucose tolerance Chinese individuals (Zhong et al., 2019). However, compared to normal glucose tolerance individuals, the abundance of metagenomic linkage groups (MLGs) from the *Clostridia* class and *Faecalibacterium prausnitzii* was lower, while that from *E. coli*, *Streptococcus salivarius*, and *Eggerthella* sp. was higher in prediabetics (Zhong et al., 2019). Meanwhile, MLGs annotated to *E. coli* were also enriched in prediabetics compared to treatment-naïve T2DM individuals. These findings indicate that different stages of T2DM show diverse characteristics of the gut microbiota, and there may be complex interactions between host and the gut microbiota.

### Gut Microbiota in T2DM Animal Models

Consistently, the association between the gut microbiota and T2DM has also been confirmed in several animal models, including mice, cats and zebrafish (Kieler et al., 2019; Okazaki et al., 2019; Wang Y. et al., 2020). Compared with normal Wistar rats, Goto-Kakizaki (GK) rats, a genetic T2DM animal model produced by repeated inbreeding of Wistar rats, showed significant enrichment of the genera *Prevotella_9*, *Blautia*, *Roseburia*, *Allobaculum* and *Prevotella_1* (Peng et al., 2019). *db/db* mouse is an animal model of spontaneous diabetes caused by leptin receptor deficiency (Suriano et al., 2021). Similarly, *db/db* mice had abnormal gut microbiota composition, and the fecal microbiota transplantation (FMT) from *db/db* mice induced increased body weight and fasting blood glucose, as well as changes in microbiota composition in pseudo-germ-free mice (Yu et al., 2019a). Moreover, Beli et al. found that certain bacterial taxa showed loss of diurnal oscillatory rhythms or phase shifts in their peak levels in *db/db* mice (Beli et al., 2019). Zucker diabetic fatty (ZDF) rat with a mutant leptin receptor is another common T2DM model, characterized by insulin resistance and metabolic disorders (Griffen et al., 2001). Of note, the relative abundance of *Ruminococcus* and *Allobaculum* was positively correlated with the levels of random blood glucose, while the relative abundance of *Lactobacillus* and *Turicibacter* was negatively correlated with the levels of random blood glucose in ZDF rats, indicating that these bacterial taxa might be implicated in the pathogenesis of T2DM (Zhou et al., 2019b). In high-fat diet (HFD) and streptozotocin (STZ) treated mice, genistein, an active isoflavone, alleviated insulin resistance and inflammation response by regulating the abundance of genera *Bacteroides*, *Prevotella*, *Helicobacter* and *Ruminococcus*, suggesting that the gut microbiota could be a potential target for the treatment of T2DM (Yang and Jia, 2021).

In addition, the abundance of several butyrate-producing bacterial genera, such as *Dialister*, *Anaerotruncus* and unknown *Ruminococcaceae*, was reduced in diabetic cats compared to healthy lean cats (Kieler et al., 2019). At the same time, the gut microbiota was related to specific clinical parameters in diabetic cats (Kieler et al., 2019). Specifically, the relative abundance of *Enterobacteriaceae* was positively correlated with serum fructosamine levels, which reflected the long-term blood glucose levels of cats, while the relative abundance of *Ruminococcaceae* was negatively correlated with the levels of serum amyloid A. Okazaki et al. established a T2DM zebrafish model by overfeeding for 4 weeks and found lower bacterial diversity in the T2DM zebrafish intestine (Okazaki et al., 2019). Intriguingly, certain members of the gut microbiota were necessary for early pancreatic β-cell development in zebrafish (Hill et al., 2016). Further studies found that some *Aeromonas* strains could secrete β-cell expansion factor A (BefA), which promoted β-cell expansion by inducing proliferation. More importantly, the specific bacterial species in humans could also secrete BefA-like proteins, and these proteins had similar functions, which provided new ideas for T2DM treatment (Hill et al., 2016). Interestingly, several bacterial taxa, such as *Bacteroides* (Zhang et al., 2013; Zhang et al., 2021b), *A. muciniphila* (Yan et al., 2016; Zhang J. et al., 2021), and *Lactobacillus* (Sato et al., 2014; Gu et al., 2016), showed consistent trends in T2DM patients and animal models. Therefore, it is of great interest to explore the molecular mechanisms by which these bacterial taxa participate in the development of diabetes.

### Gut Microbiota and Diabetic Complications

Notably, the gut microbiota is closely associated with a variety of diabetic complications, such as diabetic nephropathy (DN), diabetes-induced cognitive impairment (DCI), diabetic retinopathy (DR), and diabetic peripheral neuropathy (DPN) (Salguero et al., 2019; Jayasudha and Das, 2020; Xie et al., 2020b; Zhang et al., 2021a). DN is one of the common microvascular complications of diabetes mellitus, which eventually develops into end-stage renal failure. Previous studies have found significant differences in the composition of the intestinal microbiota between DN, T2DM without renal disease and healthy individuals (Tao et al., 2019). Specifically, the *Escherichia-Shigella* and *Prevotella_9* genera could accurately distinguish DN patients from T2DM without renal disease patients, while the *Prevotella_9* genus could accurately distinguish T2DM without renal disease patients from healthy controls. Moreover, patients with stage IV DN had a higher abundance of *Haemophilus* and *Lachnospiraceae_UCG-004* compared with patients with stage III DN, indicating that the gut microbiota was involved in the progression of DN (Du et al., 2021). Further research demonstrated that the dysregulated gut microbiota inhibited adenosine 5’-monophosphate-activated protein kinase α (AMPKα) by activating G protein-coupled receptor 43 (GPR43), which in turn led to insulin resistance-mediated podocyte and kidney damage (Lu et al., 2021). Importantly, the gut microbiota depletion induced by broad-spectrum antibiotics or the gut microbiota improvement by FMT could reduce glomerular damage in diabetic rats, indicating that the gut microbiota played a key role in the development of DN. Of note, the gut microbiota-derived metabolites, including short chain fatty acids (SCFAs), trimethylamine N-oxide (TMAO) and phenyl sulfate, are also related to the development of DN (Al-Obaide et al., 2017; Kikuchi et al., 2019; Zhao et al., 2019). For example, phenyl sulfate could predict early proteinuria in patients with DN (Kikuchi et al., 2019). In diabetic mice, phenyl sulfate induced proteinuria by promoting podocyte damage, indicating that this metabolite might be a potential target for the treatment of DN.

Diabetes predisposes to cognitive impairment and may be related to the increased incidence of dementia in elderly diabetic patients. However, the research on the pathogenesis of DCI is still in the preliminary stage. Compared to T2DM patients with normal cognition, patients with cognitive impairment showed a unique composition of intestinal microbiota, which was characterized by decreased abundance of *Bifidobacterium*, *Tenericutes* and *unrank-RF39*, as well as increased abundance of *Peptococcus* and *unrank-Leuconostocaceae* (Zhang et al., 2021a). Meanwhile, gut microbiota dysbiosis was also found in DCI animal models (Wang et al., 2018; Yu et al., 2019b). Zheng et al. discovered that vancomycin, a broad-spectrum antibiotic, reduced the levels of synaptophysin (SYP) by inhibiting acetate-producing bacteria, thereby accelerating cognitive impairment in diabetic mice (Zheng et al., 2021). Interestingly, this cognitive impairment could be reversed by FMT or exogenous acetate supplementation, indicating that acetate-producing bacteria were necessary to maintain normal cognitive functions. It is worth noting that several interventions, including intermittent fasting, synbiotics and traditional Chinese medicine (TCM), have shown certain efficacy in treating DCI (Gu et al., 2017; Liu and Dai, 2020; Morshedi et al., 2020). Intermittent fasting improved the cognitive dysfunction in *db/db* mice by reconstructing the gut microbiota and changing microbial metabolites, and the mechanism might be related to the regulation of mitochondrial biogenesis and energy metabolism in the hippocampus (Liu and Dai, 2020). These findings indicate that the gut microbiota and microbial metabolites may be ideal targets for the treatment of DCI.

The latest research pointed out that the gut microbial signatures composed of 25 bacterial families could distinguish DR patients from diabetic patients without retinopathy and healthy controls (Huang et al., 2021). Among them, the relative abundance of *Pasteurellaceae* was the lowest in DR patients, which alone could be used as a non-invasive biomarker for the diagnosis of DR. Significantly, plasma TMAO levels in DR individuals were significantly higher than those in non-retinopathy T2DM individuals, and its levels were positively correlated with the incidence of DR, indicating that TMAO might be involved in the development of DR (Liu W. et al., 2021). Xie et al. discovered that STZ-induced DPN rats showed gut microbiota dysbiosis, which was characterized by enrichment of *Klebsiella*, *Coprococcus*, *Prevotella* and other bacterial taxa (Xie et al., 2020a). Importantly, The TCM compound Jinmaitong improved the phenotype of peripheral neuropathy by regulating the gut microbiota composition and increasing the levels of neuregulin 1 (NRG1) in DPN rats. In addition, the gut microbiota was also associated with diabetic macrovascular complications and diabetes-induced reproductive system damages (Sanchez-Alcoholado et al., 2017; Tian et al., 2021; Zhu et al., 2021). Together, these data suggest that the gut microbiota and microbial metabolites play a vital role in the complications of diabetes. However, the molecular mechanisms underlying them still need to be studied in depth.

## Molecular Mechanisms Linking Host and the Gut Microbiota in T2DM

As mentioned above, there is a complex relationship between the gut microbiota and T2DM. Therefore, it is significant to understand the interaction between host and microbiota in the context of abnormal glucose metabolism. In pathological conditions, the dysregulation of host molecules can lead to changes in the composition of the gut microbiota. In turn, the microbiota plays a regulatory role to participate in the development of T2DM.

### Host Molecules That Induce Gut Microbiota Dysbiosis

**Figure 2:**
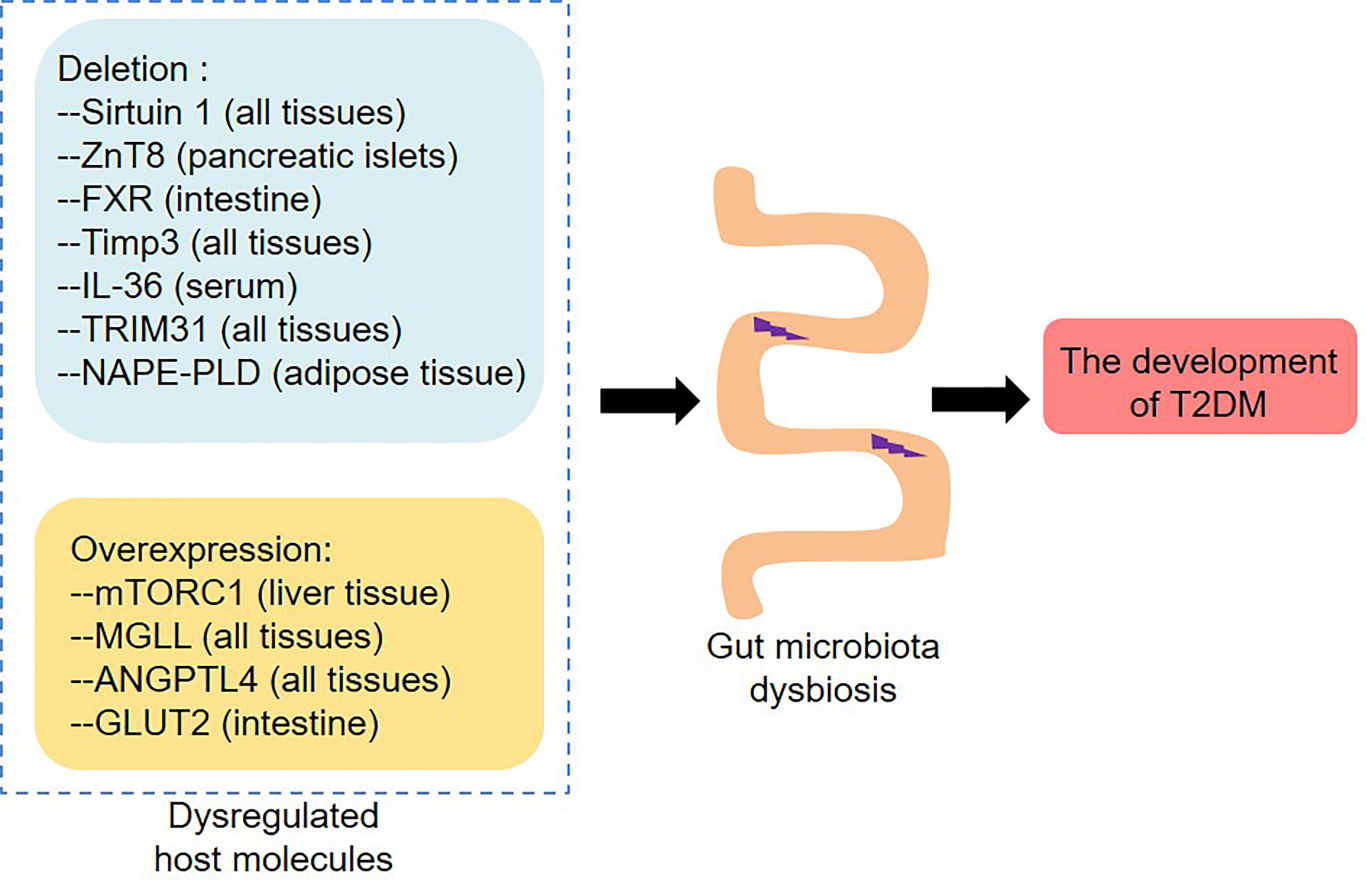
Dysregulated host molecules change the composition of the gut microbiota and thus contribute to the development of T2DM. Deletion of Sirtuin 1, ZnT8, FXR, Timp3, IL-36, TRIM31 and NABE-PLD, as well as overexpression of mTORC1, MGLL, ANGPTL4 and GLUT2, induce gut microbiota dysbiosis. ZnT8, Zinc transporter 8; FXR, Farnesoid X receptor; Timp3, Tissue inhibitor of metalloproteinase 3; IL-36, Interleukin 36; TRIM31, Tripartite motif-containing protein 31; NAPE-PLD, N-acylphosphatidylethanolamine phospholipase D; mTORC1, Mechanistic target of rapamycin complex 1; MGLL, Monoglyceride lipase; ANGPTL4, Angiopoietin-like 4; GLUT2, Glucose transporter 2.

Although T2DM is related to the dysbiosis of the gut microbiota, the reason for such dysbiosis is still unclear. Of note, gut microbial homeostasis is subject to regulation by several host molecules (**Figure 2**). Previous studies have shown that the protein deacetylase sirtuin 1 protects HFD-fed mice from metabolic disorders mainly by regulating the abundance of *Firmicutes* and *Bacteroidetes* (Caron et al., 2014). HFD-fed mice lacking sirtuin 1 exhibited adipose tissue hypertrophy, fatty liver and insulin resistance. Zinc transporter 8 (ZnT8) is a transmembrane protein enriched in pancreatic β-cells, which is involved in the development of diabetes mellitus (Lee et al., 2008; Wan et al., 2017). ZnT8 deficiency induced fat accumulation and glucose intolerance in part by regulating the intestinal morphology and composition of the gut microbiota, which might increase the risk of T2DM and obesity (Mao et al., 2019). It is worth noting that *db/db* mice treated with farnesoid X receptor (FXR) agonist fexaramine showed decreased serum cholesterol and free fatty acid levels, increased glucagon-like peptide-1 (GLP-1) secretion, as well as improved insulin sensitivity (Pathak et al., 2018). In-depth research found that fexaramine activated the Takeda G protein-coupled receptor 5 (TGR5)/GLP-1 signaling pathway by increasing the abundance of *Acetatifactor* and *Bacteroides* that produced lithocholic acid, thereby improving glucose metabolism. Mavilio et al. observed that knockout of tissue inhibitor of metalloproteinase 3 (Timp3) induced liver steatosis and glucose intolerance in HFD-fed mice (Mavilio et al., 2016). Mechanistically, loss of Timp3 led to gut microbiota dysbiosis, which further triggered systemic inflammation through interleukin 6 (IL-6) signaling. Antibiotic treatment improved metabolism and reduced inflammation, indicating that the gut microbiota mediated the role of Timp3 in regulating metabolism (Mavilio et al., 2016). Furthermore, the anti-diabetic effects of host molecules such as IL-36, tripartite motif-containing protein 31 (TRIM31) and *N*-acylphosphatidylethanolamine phospholipase D (NAPE-PLD) are also related to the gut microbiota (Geurts et al., 2015; Cheng et al., 2018; Giannoudaki and Hernandez-Santana, 2019). Overall, these host proteins can improve metabolism to a certain extent by reshaping the gut microbiota, thereby preventing obesity and T2DM.

On the contrary, certain host proteins can promote the development of T2DM and related metabolic perturbations by inducing gut microbiota dysbiosis (Jung et al., 2016; Dione et al., 2020; Patil and Arvindekar, 2021). Resveratrol was a specific inhibitor of mechanistic target of rapamycin complex 1 (mTORC1), which could improve glucose intolerance and insulin resistance in HFD-fed mice, indicating that mTORC1 played an important role in glucose homeostasis (Jung et al., 2016). Importantly, resveratrol reduced the relative abundance of *Oscillibacter*, *Clostridium* XI, *Lactococcus*, *Flavonifractor* and *Hydrogenoanaerobacterium* in HFD-fed mice, and these bacterial taxa were positively correlated with fasting blood glucose levels and HOMA2-IR index, suggesting that changes in the gut microbiota induced by mTORC1 dysregulation might be related to diabetic phenotypes. Another study found that HFD-fed monoglyceride lipase (MGLL) knockout mice showed improved glucose tolerance and decreased adiposity, and that the gut microbiota of MGLL knockout mice and wild-type mice responded differently to HFD feeding (Dione et al., 2020). For example, HFD increased the abundance of *Lactobacillus* in wild-type mice, but did not change its abundance in MGLL knockout mice. These data indicate that MGLL may be a potential target for the treatment of metabolic diseases. Moreover, loss of angiopoietin-like 4 (ANGPTL4) improved glucose tolerance and increased insulin levels in mice that were fed diets rich in unsaturated fatty acids, cholesterol and fructose (Janssen et al., 2018). *Angptl4*^−/−^ mice had a higher abundance of *Adlercreutzia*, *Lactobacillus* and *SMB53*, and a lower abundance of *Allobaculum*, and the gut microbiota inhibition by antibiotics reduced the glucose tolerance in *Angptl4*^−/−^ mice, indicating that the effect of ANGPTL4 on glucose metabolism might depend on the regulation of the gut microbiota. Analogously, the gut-specific deletion of glucose transporter 2 (GLUT2) might improve the glucose homeostasis by changing the composition of the gut microbiota in mice (Schmitt et al., 2017). Together, blocking mTORC1, MGLL, ANGPTL4 or GLUT2 by drugs may be a therapeutic strategy for T2DM.

### Immune and Inflammatory Responses

**Figure 3:**
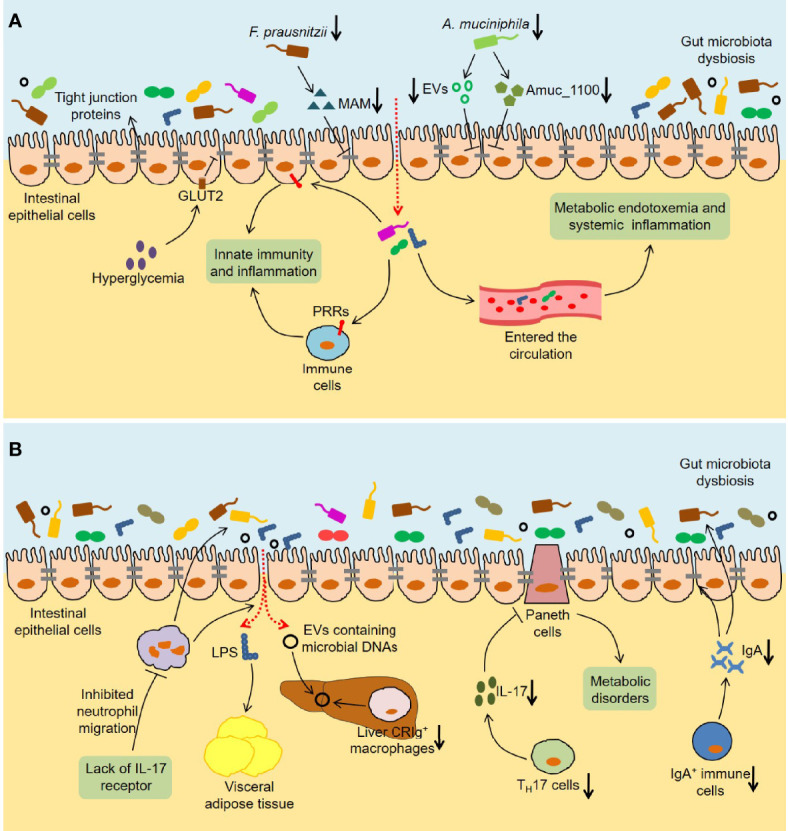
Immune and inflammatory responses induced by the gut microbiota in T2DM. **(A)** The intestinal barrier is disturbed in T2DM. Hyperglycemia disrupts the integrity of tight and adherent junctions by GLUT2. *F. prausnitzii* abundance and its metabolite MAM levels are downregulated in *db/db* mice, which results in damaged intestinal barrier by reducing ZO-1 expression. Analogously, *A. muciniphila* abundance and the levels of *A. muciniphila*-derived EVs and Amuc_1100 are reduced in diabetic mice, which also disrupts intestinal barrier integrity. The increased intestinal permeability leads to the translocation of bacteria and bacterial products, which not only activates the innate immunity and inflammation in the gut, but also causes metabolic endotoxemia and systemic inflammation. **(B)** Multiple immune cells are involved in the influence of the gut microbiota on host metabolism. Lack of IL-17 receptor promotes intestinal dysbiosis and LPS translocation into visceral adipose tissue by inhibiting the migration of neutrophils in the intestinal mucosa. Reduced liver CRIg^+^ macrophages leads to an accumulation of EVs containing gut microbial DNAs in obesity, thereby promoting tissue inflammation and insulin resistance. The number of T_H_17 cells is reduced in the intestinal tissues of obese mice, which leads to metabolic disorders by regulating the functions of Paneth cells. Furthermore, HFD-fed mice show a decrease in IgA^+^ immune cell percentage and secretory IgA concentrations in colon tissues, which impairs glucose metabolism by increasing intestinal permeability and changing microbial composition. GLUT2, Glucose transporter 2; MAM, Microbial anti-inflammatory molecule; ZO-1, Zonula occludens-1; EVs, Extracellular vesicles; PRRs, Pattern recognition receptors; IL-17, Interleukin 17; LPS, Lipopolysaccharide; CRIg, Complement receptor of the immunoglobulin superfamily.

It is reported that the crosstalk between the gut microbiota and host regulates local or systemic immunity and inflammation, which in turn contributes to the development of T2DM (Tilg and Moschen, 2014). In this process, the intestinal barrier function plays an important role (**Figure 3A**). The intestinal barrier protects the body from intestinal contents, and its dysfunction increases the leakage of bacteria or bacterial products and thus leads to chronic inflammation and metabolic diseases (Scheithauer et al., 2020). *In vivo* and *in vitro* studies have confirmed that hyperglycemia increases intestinal barrier permeability by changing the integrity of tight and adherence junctions, resulting in systemic influx of microbial products (Thaiss et al., 2018). Importantly, the lack of GLUT2 in intestinal epithelial cells maintained barrier integrity by retaining zonula occludens-1 (ZO-1) and E-cadherin, indicating that GLUT2 might be a target for improving intestinal-related inflammation and infection. In addition, the gut microbiota is also involved in the regulation of intestinal permeability in obesity and diabetes (Everard et al., 2013; Xu et al., 2020). One of the mechanisms is that the gut microbiota affects the intestinal barrier function by regulating the secretion of GLP-2 during diabetes (Cani et al., 2009; Zhang et al., 2020). Moreover, microbial anti-inflammatory molecule (MAM), a metabolite produced by *F. prausnitzii*, restored the damaged intestinal barrier by increasing ZO-1 expression in *db/db* mice (Xu et al., 2020). The abundance of *A. muciniphila* was reduced in the T2DM mouse model, and *A. muciniphila* treatment could improve metabolic endotoxemia and glucose metabolism by restoring mucus layer thickness (Everard et al., 2013). Interestingly, *A. muciniphila*-derived extracellular vesicles (EVs) reduced intestinal permeability by promoting the expression of tight junction proteins and thus improved glucose homeostasis in diabetic mice (Chelakkot et al., 2018). Another research found that the protein Amuc_1100 derived from the outer membrane of *A. muciniphila* maintained the integrity of the intestinal barrier by interacting with toll-like receptor 2 (TLR2), thereby alleviating obesity and insulin resistance in HFD-fed mice (Plovier et al., 2017). These data indicate that *A. muciniphila* is a beneficial gut bacteria and plays an important role in controlling intestinal permeability.

It is well known that innate immunity exists in the organism as a rapid anti-infection effect, and the interaction of pattern recognition receptors (PRRs) and microbe-associated molecular patterns (MAMPs) initiates this process. Invasive bacteria and bacterial products could induce inflammation in the intestinal tract by triggering innate immunity. Notably, some PRRs, namely TLRs and nucleotide-binding oligomerization domain (NOD)-like receptors (NLRs), are highly related to metabolic diseases (Winer et al., 2016). Previous studies found that TLR5-deficient mice exhibited insulin resistance and obesity, which was related to changes in the gut microbiota composition (Vijay-Kumar et al., 2010). Importantly, transplantation of the gut microbiota from TLR5-deficient mice caused metabolic syndrome in wild-type germ-free mice, suggesting that the crosstalk between TLR5 and the gut microbiota might play an important role in metabolic disease. Similarly, TLR2 knockout mice had differences in the gut microbiota compared with wild-type mice, and exhibited glucose intolerance and insulin resistance (Guadagnini et al., 2019). Another study discovered that myeloid differentiation primary-response gene 88 (MyD88), a TLR adapter, could regulate glucose and lipid metabolism in part by altering the composition of the gut microbiota (Duparc et al., 2017). The activation of NOD1 contributed to the inflammation and insulin intolerance caused by HFD (Schertzer et al., 2011). Conversely, bacterial cell wall-derived muramyl dipeptide promoted the expression of interferon regulatory factor 4 (IRF4) through NOD2, thereby improving inflammation and insulin resistance in obese mice (Cavallari et al., 2017). Additionally, increased intestinal permeability causes more bacteria and bacterial products to cross the barrier and enter the systemic circulation. Cani et al. observed that HFD-fed mice showed an increase in the proportion of lipopolysaccharide (LPS)-containing bacteria in the intestine and plasma LPS levels (Cani et al., 2007). Meanwhile, they simulated the metabolic impairment and inflammation induced by a HFD in mice through continuous subcutaneous infusion of LPS, indicating that LPS in the systemic circulation might contribute to the development of T2DM. Consistently, antibiotic treatment improved endotoxemia, inflammation, and metabolic parameters in both HFD-fed and *ob/ob* mice, indicating that the gut microbiota dysbiosis regulated intestinal permeability and further led to metabolic endotoxemia (Cani et al., 2008).

It is worth noting that various immune cells are involved in the role of the gut microbiota in host metabolism (**Figure 3B**). The lack of IL-17 receptor might promote intestinal dysbiosis and LPS translocation into visceral adipose tissue by inhibiting the migration of neutrophils in the intestinal mucosa, leading to glucose intolerance and insulin resistance in HFD-fed mice (Pérez et al., 2019). Complement receptor of the immunoglobulin superfamily (CRIg) is expressed on human macrophages and plays an important role in immune responses (Duan et al., 2021). Luo et al. observed an accumulation of EVs containing gut microbial DNAs and a decrease in the number of liver CRIg^+^ macrophages in obesity (Luo et al., 2021). Significantly, CRIg^+^ macrophages could clear these EVs in a complement C-dependent manner, thereby improving tissue inflammation and insulin resistance. Furthermore, the crosstalk between CD4^+^ T cells and the gut microbiota is closely related to the development of T2DM. On the one hand, the gut microbiota dysbiosis induced by HFD led to the decrease of T_H_17 cells in the lamina propria of the small intestine and thus promoted the occurrence of T2DM (Garidou et al., 2015). On the other hand, the number of T_H_17 cells was significantly reduced in the intestinal tissues of obese mice, and the further reduction of T_H_17 cells by a HFD lacking vitamin A aggravated glucose intolerance and insulin resistance (Hong et al., 2017). In-depth research found that T_H_17 cells might regulate the functions of Paneth cells and metabolism-related intestinal microbiota through IL-17, thereby maintaining metabolic homeostasis (Hong et al., 2017). Luck et al. discovered that HFD-fed mice showed a decrease in IgA^+^ immune cell percentage and secretory IgA concentrations in colon tissues, and that glucose metabolism disorders were more serious in HFD-fed IgA-deficient mice (Luck et al., 2019). Importantly, IgA could improve glucose metabolism by regulating intestinal permeability and microbial compositions, indicating that the treatments targeting IgA^+^ immune cells might have therapeutic value for T2DM.

### Gut Microbial Metabolites

**Figure 4:**
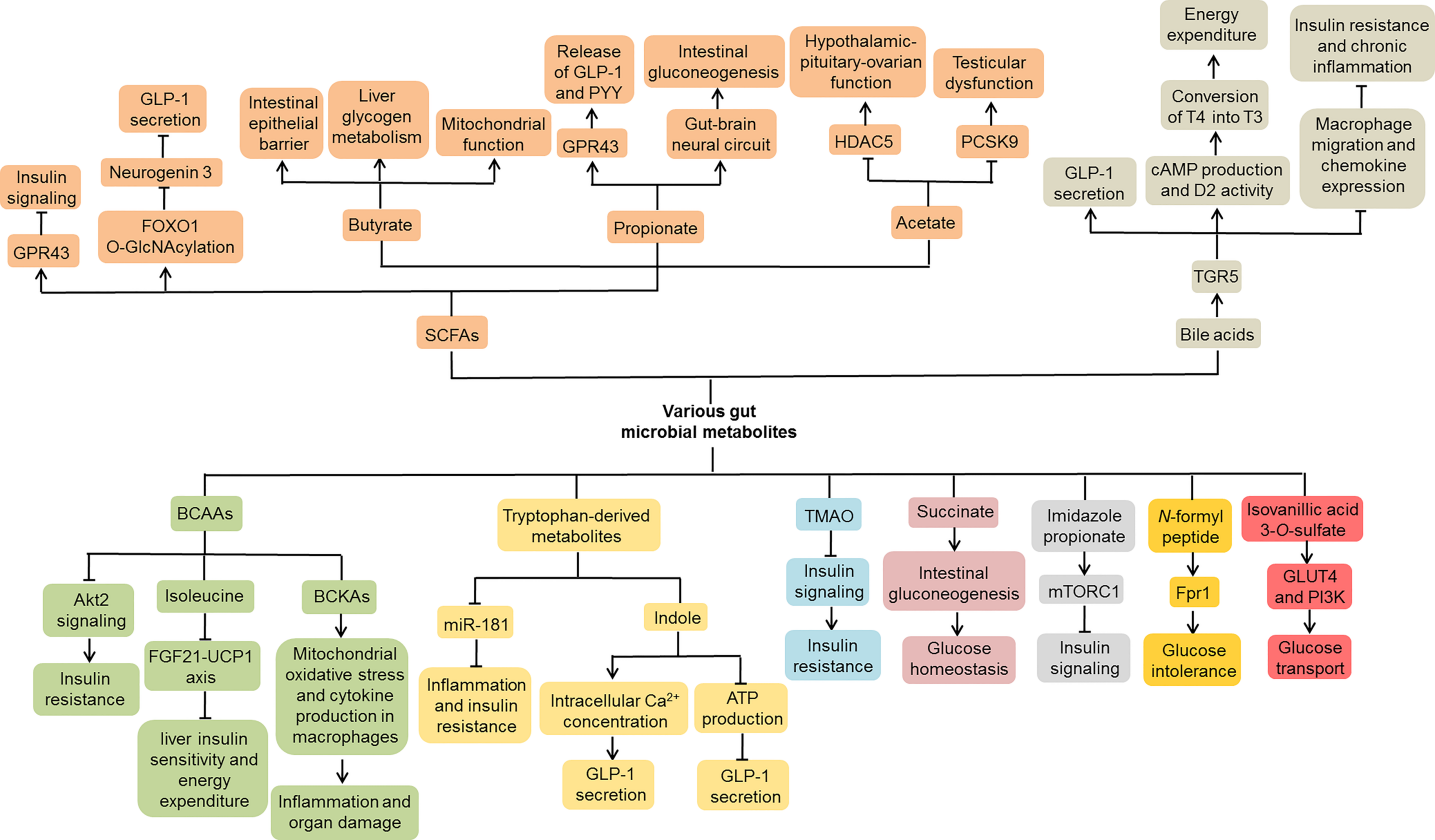
Molecular mechanisms of gut microbial metabolites involved in T2DM. Various gut microbial metabolites, including SCFAs, bile acids, BCAAs, tryptophan-derived metabolites, TMAO, succinate, imidazole propionate, *N*-formyl peptide and isovanillic acid 3-*O*-sulfate, contribute to the development of T2DM through complex molecular mechanisms. SCFAs, Short chain fatty acids; BCAAs, Branched-chain amino acids; TMAO, Trimethylamine N-oxide; GPR43, G protein-coupled receptor 43; FOXO1, Forkhead box O1; GLP-1, Glucagon-like peptide-1; PYY, Peptide YY; HDAC5, Histone deacetylase-5; PCSK9, Proprotein convertase subtilisin/kexin type 9; TGR5, Takeda G-protein receptor 5; cAMP, Cyclic AMP; D2, Type 2 iodothyronine deiodinase; FGF21, Fibroblast growth factor 21; UCP1, Uncoupling protein 1; BCKAs, Branched-chain α-keto acids; mTORC1, Mechanistic target of rapamycin complex 1; GLUT4, Glucose transporter 4; PI3K, Phosphoinositide 3-kinase.

Conspicuously, various gut microbial metabolites, such as SCFAs, TMAO and tryptophan-derived metabolites, have been reported to be closely related to the pathogenesis of T2DM (Jia et al., 2019; Mercer et al., 2020; Noureldein et al., 2020) (**Figure 4**). Previous studies found that SCFAs inhibited insulin signaling by activating G protein-coupled receptor 43 (GPR43) in adipocytes, thereby suppressing fat accumulation in adipose tissue and promoting energy metabolism in other tissues (Kimura et al., 2013). In addition, SCFAs inhibited the expression of Neurogenin 3 through forkhead box O1 (FOXO1) O-GlcNAcylation and thus suppressed L cell development (Zhao M. et al., 2020). The protein O-GlcNAcylation deficiency in intestinal epithelial cells promoted L cell hyperplasia and GLP-1 secretion in mice, thereby improving glucose metabolism. These findings indicate that SCFAs may be involved in the development of T2DM. Among them, butyrate is one of the well-studied SCFAs produced by the gut microbiota. It could ameliorate the progression of T2DM *via* a variety of mechanisms, including maintaining the integrity of the intestinal epithelial barrier (Xu et al., 2018), promoting liver glycogen metabolism (Zhang et al., 2019), as well as regulating the function of mitochondria (Zhao T. et al., 2020). Another SCFA propionate promoted the release of GLP-1 and peptide YY (PYY) in rats and mice by activating GPR43 (Psichas et al., 2015). Likewise, acute supplementation of propionate increased postprandial plasma levels of these two gut hormones in humans, and its long-term supplementation prevented weight gain and worsening insulin sensitivity in overweight adults (Chambers et al., 2015). Moreover, propionate activated intestinal gluconeogenesis by the GPR41-dependent gut-brain neural circuit, thereby maintaining glucose and energy homeostasis (De Vadder et al., 2014). It is worth noting that acetate is related to diabetes-associated reproductive abnormalities. Acetate not only improved the hypothalamic-pituitary-ovarian function of female patients with T2DM by inhibiting histone deacetylase-5 (HDAC5) (Olaniyi et al., 2021a), but also alleviated the testicular dysfunction in male patients with diabetes by inhibiting proprotein convertase subtilisin/kexin type 9 (PCSK9) (Olaniyi et al., 2021b). Collectively, SCFAs, such as acetate, propionate and butyrate, have played an important role in the development of T2DM.

The primary bile acids are products of cholesterol metabolism in the liver, which are involved in the absorption and transport of lipids and fat-soluble vitamins. When primary bile acids are secreted into the intestine, they are metabolized by the gut microbiota into secondary bile acids through deconjugation and 7-dehydroxylation (Yesair and Himmelfarb, 1970; Devlin and Fischbach, 2015; Wahlström et al., 2016). It is well known that most secondary bile acids promote the secretion of GLP-1 from intestinal L cells mainly by activating Takeda G-protein receptor 5 (TGR5) (Katsuma et al., 2005; Thomas et al., 2009; Chaudhari et al., 2021). Moreover, bile acids could improve metabolic control by inducing energy expenditure in adipose tissue (Watanabe et al., 2006). Mechanistically, bile acids increased cyclic AMP (cAMP) production and type 2 iodothyronine deiodinase (D2) activity by interacting with TGR5, thereby promoting the conversion of inactive thyroxine (T4) into active thyroid hormone (T3). Interestingly, bile acid-induced activation of TGR5 ameliorated insulin resistance and chronic inflammation in obese mice, which was related to macrophage migration and chemokine expression in macrophages (Perino et al., 2014). These data indicate that bile acids participate in the pathogenesis of T2DM by regulating energy metabolism and inflammation response.

Growing evidence suggests that individuals with diabetes or insulin resistance exhibit increased levels of branched-chain amino acids (BCAAs) (Pedersen et al., 2016; Vanweert et al., 2021). In HFD-fed mice, BCAAs aggravated liver insulin resistance by inhibiting Akt2 signaling, that is, increasing liver gluconeogenesis and inhibiting liver adipogenesis (Zhao H. et al., 2020). Of note, a low-isoleucine diet increased liver insulin sensitivity and energy expenditure by activating the fibroblast growth factor 21 (FGF21)-uncoupling protein 1 (UCP1) axis in mice, while a low-leucine diet had no such effects, indicating that each BCAA might have different contributions to host metabolism (Yu et al., 2021). Normally, BCAAs are metabolized into branched-chain α-keto acids (BCKAs) by branched-chain aminotransferases (BCATs), which are further irreversibly decarboxylated by branched-chain α-ketoacid dehydrogenase (BCKDH) complex (Lynch and Adams, 2014). *db/db* mice showed decreased activity of BCKDH complex and increased levels of BCKAs (Liu S. et al., 2021). In-depth studies found that BCKAs could induce mitochondrial oxidative stress and cytokine production in macrophages, thereby exacerbating inflammation and organ damage in T2DM (Liu S. et al., 2021).

Virtue et al. observed that microbial metabolites of tryptophan, such as indole and indoxyl sulfate, slowed down the progress of inflammation and insulin resistance by inhibiting the expression of miR-181 in white adipose tissue (Virtue et al., 2019). Intriguingly, indole could regulate GLP-1 secretion from L cells through a complex mechanism (Chimerel et al., 2014). On the one hand, indole acutely promoted GLP-1 secretion by increasing the intracellular Ca^2+^ concentration. On the other hand, long-term indole stimulation reduced GLP-1 secretion by inhibiting the production of ATP. These results suggest that tryptophan-derived metabolites may serve as important signal molecules that affect host metabolism. Nutrients containing trimethylamine are metabolized by gut microbiota to form trimethylamine, which is then converted into TMAO in the liver. Higher plasma TMAO concentrations were associated with an increased risk of T2DM (Shan et al., 2017). TMAO could block insulin signaling in the liver and induce inflammation in adipose tissues, thus aggravating insulin resistance in HFD-fed mice (Gao et al., 2014). Additionally, other microbiota-produced metabolites, such as succinate, imidazole propionate, *N*-formyl peptide and isovanillic acid 3-*O*-sulfate, are also related to host glucose metabolism, which may provide new targets for T2DM treatment strategies (De Vadder et al., 2016; Koh et al., 2018; Houghton et al., 2019; Wollam et al., 2019).

### Other Mechanisms

Notably, the gut microbiota composition in elderly T2DM and periodontitis patients has changed compared with healthy controls and elderly patients with T2DM alone, especially the increase in the abundance of *Prevotella copri* and the decrease in the abundance of *F. prausnitzii*, indicating that the gut microbiota may mediated the influence of periodontitis on diabetes (Li J. et al., 2020). In C57BL/6J mice, periodontitis induced alterations in the gut microbiota and high levels of fasting blood glucose and glucose intolerance, while the glucose metabolism was improved after antibiotic treatment (Li et al., 2021). Further research found that *Porphyromonas gingivalis* exacerbated fasting and postprandial hyperglycemia by inducing changes in the gut microbiota in *db/db* mice, which might be related to excessive hepatic gluconeogenesis (Kashiwagi et al., 2021). Grasset et al. observed that GLP-1-induced insulin secretion and gastric emptying were disrupted in HFD-fed mice (Grasset et al., 2017). Mechanistically, the dysregulated gut microbiota damaged the GLP-1-activated gut-brain axis through reducing the nitric oxide production by enteric neurons and thus led to GLP-1 resistance. Furthermore, the relative abundance of *Bacteriodes uniformis* and *Phascolarctobacterium faecium* was decreased in T2DM patients and was negatively correlated with insulin and fasting blood glucose (Li L. et al., 2020). Importantly, their abundance was negatively correlated with serum miR-122-5p levels, indicating that the interaction between the gut microbiota and miRNAs might be involved in the development of T2DM.

## Conclusion and Future Perspective

As mentioned above, the gut microbiota plays an important role in the development of T2DM, which greatly expands our understanding of the pathogenesis of this metabolic disease. Meanwhile, accumulating evidence links changes in the gut microbiota with T1DM, gestational diabetes and latent autoimmune diabetes in adults (LADA), indicating that the gut microbiota may be a key regulator of host glucose metabolism and immune response (Zheng et al., 2020; Fang et al., 2021; Qin et al., 2021). However, most of the research focuses on gut bacteria, and little attention is paid to viruses and fungi in the intestinal tract. Recent studies found that the diversity of gut viruses in obese T2DM patients was reduced compared with lean controls (Yang et al., 2021). Moreover, the fungal communities of T2DM patients were different from those of healthy individuals, showing high abundance of *Malessezia firfur* and unclassified *Davidiella* as well as low abundance of unclassified *Basidiomycota* (Al Bataineh et al., 2020). These findings indicate that the gut microbiota is a complex ecosystem and is inextricably linked to T2DM. Nevertheless, further studies are needed to explore which specific microbes are responsible for diabetes physiopathology and the molecular mechanisms at work.

**Figure 5:**
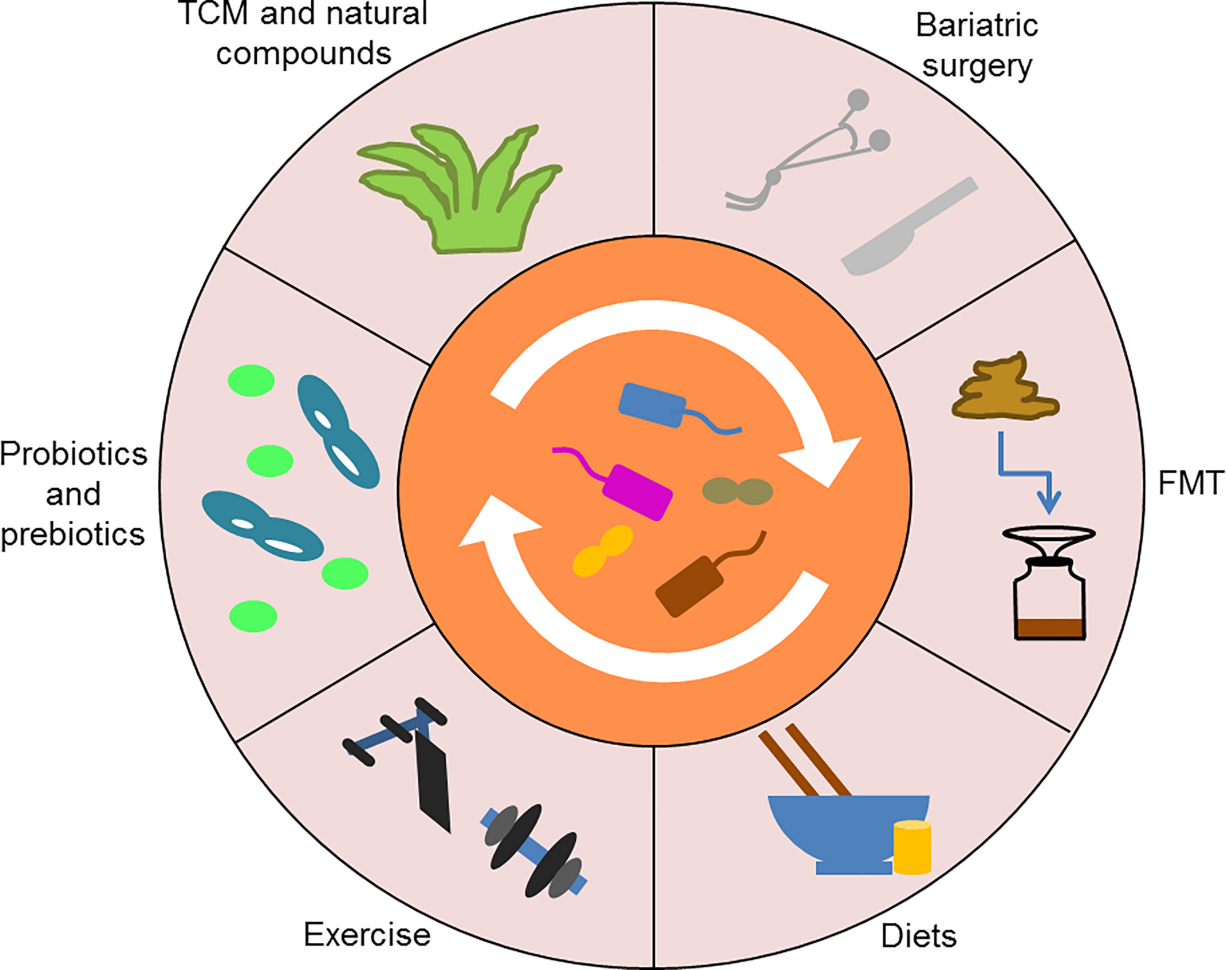
Various antidiabetic interventions targeting the gut microbiota. Probiotics and prebiotics, TCM and natural compounds, as well as certain non-drug therapies, such as bariatric surgery, FMT, diets, and exercise, can reshape the gut microbiota to treat T2DM. TCM, Traditional Chinese medicine; FMT, Fecal microbiota transplantation.

Significantly, various antidiabetic interventions targeting the gut microbiota are being explored (**Figure 5**). Among them, the most promising ones for clinical application are probiotics and prebiotics (Lee Y. et al., 2021; Watanabe et al., 2021). *Lactobacillus rhamnosus* LRa05 reduced fasting blood glucose and insulin resistance by regulating the composition of the gut microbiota in T2DM mice (Wu et al., 2021). Chang et al. demonstrated that the polysaccharides derived from *Ganoderma lucidum* improved insulin resistance and inflammation in HFD-fed mice by reversing the gut microbiota dysbiosis and maintaining intestinal barrier integrity, indicating that the polysaccharides might be an effective prebiotic for diabetes treatment (Chang et al., 2015). In addition, TCM and natural compounds show great potential for restoring homeostasis in the intestinal microenvironment. For example, ginsenoside Rg5 could modulate the abundance of multiple bacterial taxa, such as *Firmicutes*, *Verrucomicrobia* and *Bacteroidetes*, in *db/db* mice and thus exert anti-diabetic effects (Wei et al., 2020). Of note, certain non-drug therapies, such as bariatric surgery, FMT, diets and exercise, can also slow the progression of T2DM by reshaping the gut microbiota (Wei et al., 2018; Liu et al., 2020; Wang C. et al., 2020; Ng et al., 2021). In conclusion, the gut microbiota is closely related to glucose metabolism, which may provide novel ideas for the treatment of T2DM.

## Author Contributions

ZZ and BS contributed to drafting and revising the article. DY and CZ contributed to the conception and design. All authors approved the final version.

## Funding

This work was supported by funding from the National Natural Science Foundation of China (No. 82104307) and the Natural Science Foundation of Hunan Province (No. 2021JJ40865).

## Conflict of Interest

The authors declare that the research was conducted in the absence of any commercial or financial relationships that could be construed as a potential conflict of interest.

## Publisher’s Note

All claims expressed in this article are solely those of the authors and do not necessarily represent those of their affiliated organizations, or those of the publisher, the editors and the reviewers. Any product that may be evaluated in this article, or claim that may be made by its manufacturer, is not guaranteed or endorsed by the publisher.
